# *Lactobacillus plantarum* ZS2058 and *Lactobacillus rhamnosus* GG Use Different Mechanisms to Prevent *Salmonella* Infection *in vivo*

**DOI:** 10.3389/fmicb.2019.00299

**Published:** 2019-02-20

**Authors:** Junsheng Liu, Zhennan Gu, Fanfen Song, Hao Zhang, Jianxin Zhao, Wei Chen

**Affiliations:** ^1^State Key Laboratory of Food Science and Technology, Jiangnan University, Wuxi, China; ^2^School of Food Science and Technology, Jiangnan University, Wuxi, China; ^3^National Engineering Research Center for Functional Food, Jiangnan University, Wuxi, China; ^4^Beijing Innovation Centre of Food Nutrition and Human Health, Beijing Technology and Business University, Beijing, China

**Keywords:** *Lactobacillus*, *Salmonella*, preventive effect, different, mechanism

## Abstract

Pathogen-induced infectious diseases pose great threats to public health. Accordingly, many studies have investigated effective strategies targeting pathogenic infections. We previously reported the preventive effects of *Lactobacillus plantarum* ZS2058 (ZS2058) and *L. rhamnosus* GG (LGG) against *Salmonella* spp. in a murine model. Here, we compared the mechanisms underlying the preventive effects of these *Lactobacillus* strains *in vivo*. Notably, reduced C-reactive protein levels were observed with both ZS2058 and LGG, which suggests abrogated anti-infection and inflammatory responses. ZS2058 more efficiently reduced the pathogenicity of *Salmonella* by increasing the level of propionic acid in feces and production of mucin 2 in the mouse colon and activity through the interleukin (IL)-23/IL-22 and IL-23/IL-17 pathways. Meanwhile, LGG more strongly alleviated gut inflammation, as indicated by changes in the levels of tissue necrosis factor (TNF)-α, IL-10 and myeloperoxidase (MPO) in infected mice. Moreover, both ZS2058 and LGG restored the levels of interferon (INF)-γ, a cytokine suppressed by *Salmonella*, albeit through different pathways. Our results demonstrate that ZS2058 and LGG prevent *Salmonella* infection via different mechanisms.

## Introduction

*Salmonella* infection, or salmonellosis, is associated with high morbidity and mortality and is therefore a significant public health concern worldwide. This problem is much more severe in developing countries because of the presence of contaminated food and water and poor sanitation facilities (Castillo et al., 2012). Therefore, investigations of effective strategies for coping with infectious diseases are highly significant.

Salmonellosis is usually treated clinically with antibiotics. However, these drugs can cause side effects, including antibiotic resistance and enteric dysbacteriosis. To date, some strains of multi-antibiotic-resistant *Salmonella enterica* serovar Typhimurium (*S.* Typhimurium) have been identified in poultry (Rajashekara et al., 2000). Other work has found that antibiotic therapy can exacerbate *Salmonella*-induced diarrhea and increase the period of pathogen shedding by at least 3 weeks (Neill et al., 1991). Accordingly, novel and safe strategies for salmonellosis prevention are vitally important.

Probiotics have been identified as a highly promising alternative treatment option for *Salmonella* infection because these products are associated with fewer side effects and better safety. Many studies have therefore investigated the ability of probiotics to prevent *Salmonella* infection, as well as the involved mechanisms. Several *Lactobacillus* strains were found to attenuate the intestinal epithelial barrier dysfunction induced by *Salmonella* lipopolysaccharide (LPS) (Yeung et al., 2013). *L. rhamnosus* S1K3 promotes the transcription of genes encoding Toll-like receptors in Peyer’s patches (PPs) and modulates the levels of cytokines, which ultimately decreases the *Salmonella* load in mouse fecal matter and prevents bacterial invasion of internal organs (Kemgang et al., 2016). In a chicken model, *L. salivarius* CTC2197 was found to reduce *Salmonella* colonization (Pascual et al., 1999). Furthermore, the transcription of virulence genes identified as important contributors in *Salmonella* infection were reported to be modulated by *Bifidobacterium thermophilum* RBL67. This might indicate an important mechanism that could be targeted by probiotics to reduce pathogenicity and promote pathogen clearance (Tanner et al., 2016).

*L. rhamnosus* GG (LGG) is a well-established and widely recognized probiotic strain used extensively in scientific research and clinical applications. This strain exhibits strong antimicrobial activity against *S.* Typhimurium via the accumulation of lactic acid (De Keersmaecker et al., 2006). In a C3H/He/Oujco mouse model of infection, LGG reduces the population of *S.* Typhimurium, as well as the associated mortality (Hudault et al., 1997). Our previous studies demonstrated that both *L. plantarum* (*L. plantarum*) ZS2058 and LGG exhibited strong preventive effects against *Salmonella*-induced animal death in a mouse model (Liu et al., 2018). In this study, we investigated and compared the mechanisms by which ZS2058 and LGG prevent *Salmonella* infection. Notably, we found that ZS2058 and LGG each used several distinct pathways to prevent *Salmonella* infection.

## Materials and Methods

### Bacteria and Culture Conditions

*L. plantarum* ZS2058 (ZS2058) and T (T), LGG and *S.* Typhimurium SL1344 (SL1344) were obtained from the Culture Collections of Food Microbiology (CCFM) at Jiangnan University (Wuxi, China). Lactobacilli and salmonellae were cultured, respectively, in MRS and LB broth (0.3 M NaCl) at 37°C unless otherwise stated.

### Animal Experiments

Specific pathogen-free mice (SPF; C57BL/6, female, age: 6–8 weeks) were obtained from the Model Animal Research Center of Nanjing University and housed in a controlled room (SPF, constant temperature of 22°C ± 2°C and humidity of 55% ± 5%) with a 12 h light-dark cycle at the Animal Experiment Center of Jiangnan University. This study was carried out in accordance with the recommendations of the European Community guidelines (Directive 2010/63/EU). The protocol was approved by the Ethics Committee of Jiangnan University.

Lactobacilli were washed with phosphate-buffered saline (PBS) and resuspended to a density of 5.0 × 10^9^ CFU/ml. Each mouse was administered 0.1 ml of bacterial suspension or PBS (control and infection model groups) via gavage for 10 days. Subsequently, the mice were infected with 1.0 × 10^6^ CFU of *S.* Typhimurium SL1344 (Liu et al., 2018). At 2 day post-infection, the mice were sacrificed and samples of sera and intestinal tissue were collected and stored properly (*n* = 5 or 6).

To study the potential effects of lactobacilli on SCFAs production, *in vivo* experiments were designed to analyze SCFAs changes in feces of healthy mice. Mice received PBS (control group) or lactobacilli for 10 days (*n* = 5) without *Salmonella* infection. Feces were collected at different times (see Table 1) and the content of SCFAs were analyzed.

**Table 1:** Analysis of short chain fatty acids (SCFAs) in mice feces^a^.

SCFAs, μmol/g	Group	10 days of gavage	7 days post-gavage	14 days post-gavage
Acetic acid	Control	77.01 ± 3.58	73.70 ± 5.72	65.53 ± 5.51
	ZS2058	92.72 ± 9.89	86.37 ± 6.08	70.29 ± 4.48
	T	97.33 ± 4.85	73.99 ± 4.92	79.54 ± 11.71
	LGG	81.78 ± 8.90	68.52 ± 3.83	80.55 ± 12.24
Propionic acid	Control	10.66 ± 0.54	11.59 ± 0.77	11.76 ± 1.19
	ZS2058	18.63 ± 2.00^∗^	15.32 ± 5.42	10.22 ± 0.91
	T	12.91 ± 3.74	12.59 ± 3.64	10.01 ± 0.76
	LGG	13.40 ± 4.06	10.05 ± 1.34	12.68 ± 0.78
Butyric acid	Control	7.03 ± 1.66	6.45 ± 1.29	6.81 ± 0.86
	ZS2058	8.31 ± 2.11	9.60 ± 1.58	7.65 ± 2.02
	T	6.24 ± 2.06	7.69 ± 2.77	6.99 ± 2.77
	LGG	7.77 ± 1.98	5.70 ± 1.55	5.82 ± 2.34


*^*a*^Data were analyzed by using T-test, n = 5. ^∗^p < 0.05 vs. Control.*

### C-Reactive Protein (CRP) Determination

The serum levels of CRP were measured using enzyme-linked immunosorbent assay (ELISA) kits (Nanjing Senbeijia Biological Technology Co., Ltd., China).

### SCFAs Concentration in Feces

Fecal concentrations of SCFAs were measured as previously reported (Li et al., 2016). Briefly, feces were collected, weighed and freeze-dried. Subsequently, the fecal matter was soaked in saturated sodium chloride and treated with aqueous sulfuric acid and diethyl ether for acidification and extraction, respectively. The SCFA analysis was performed via gas chromatography-mass spectrometry on a GCMS-QP2010 Ultra device (Shimadzu Co., Tokyo, Japan).

### Determination of the Levels of Mucin 2 (MUC2), Myeloperoxidase (MPO), and Cytokines

The stored samples of intestines were homogenized at a 1:9 (m/v) dilution in cold PBS. The levels of MUC2, MPO and various cytokines [tissue necrosis factor (TNF)-α, interleukin (IL)-23, IL-22, IL-17, IL-10, interferon (IFN)-γ, IL-18, IL-2, IL-1α, IL-12, and transforming growth factor (TGF)-β] in these homogenates and the stored serum samples were then determined using ELISA kits (Nanjing Senbeijia Biological Technology Co., Ltd.).

### Body, Spleen and Liver Weights, and Organ Relative Ratios

Body weights (BWs) and organ weights were obtained at different time points or at dissection. The organ relative ratio was calculated as the g/g BW (Yoshimi et al., 2001).

### Statistics

Data are expressed as means ± standard errors of the means. Mean values of different groups were analyzed using a one-way variance analysis (one-way ANOVA) with Duncan’s multiple range tests with SPSS 16.0 Statistical Software (IBM Corporation, Armonk, NY, United States). Data were considered to be statistically different at a *P* < 0.05, and were indicated by different superscript letters (such as a, b, and c). Differences between means that do not share a letter are statistically significant.

## Results

### Both ZS2058 and LGG Reduced CRP Levels in Infected Mice

**FIGURE 1:**
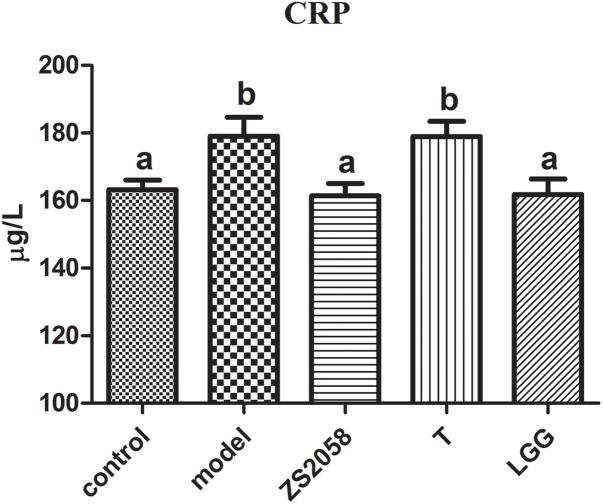
Effects of ZS2058 and LGG on CRP levels in mouse sera. Mice were pretreated with *L. plantarum* ZS2058 (ZS2058), T, *L. rhamnosus* GG (LGG) or phosphate-buffered saline (PBS) and subsequently infected with *Salmonella* Typhimurium SL1344. C-reactive protein (CRP) levels in sera were measured. The statistical difference of different groups was analyzed by one-way ANOVA, and were considered to be significantly different when *p*-value was < 0.05 (indicated by different superscript letters).

An infection is an important source of inflammatory stimuli and thus promotes the production of CRP (McDade et al., 2008), which can then be used clinically as a diagnostic parameter for infection and bacterial sepsis. As shown in Figure 1, *Salmonella*-infected mice exhibited significantly increased CRP levels (179.1 vs. 163.2 μg/L in the uninfected group). Both ZS2058 (161.4 μg/L) and LGG treatments (161.8 μg/L) were associated with reduced levels of CRP, indicating an alleviation of *Salmonella* infection *in vivo*. Our results demonstrate that pretreatment with either *Lactobacillus* strain could reduce CRP levels in the sera of infected mice.

### ZS2058 Showed More Efficiency in Reducing the Pathogenicity of *Salmonella* in Intestinal Phase

#### ZS2058 Increased Propionic Acid Level in Feces

The increased research interest in probiotics and prebiotics has directed increasing levels of attention toward SCFAs. As shown in Table 1, PA levels in fecal matter were significantly increased after a 10 days course of ZS2058 gavage (18.63 μmol/g) when compared with the PBS-treated (control) group (10.66 μmol/g). Other treatments did not induce any significant changes in the SCFA profile at any time post-gavage (Table 1). At 7 and 14 days post-gavage, no changes in the tested SCFAs were observed after treatment with ZS2058, T or LGG. Our results therefore demonstrate that ZS2058 more strongly affected PA production. As this SCFA has been reported to limit *Salmonella* colonization *in vivo* (Alshawabkeh and Tabbaa, 2002), our finding suggests that ZS2058 might protect the host from infection by increasing the production of PA.

#### ZS2058 Reduced the Level of MUC2 in Infected Mice

**FIGURE 2:**
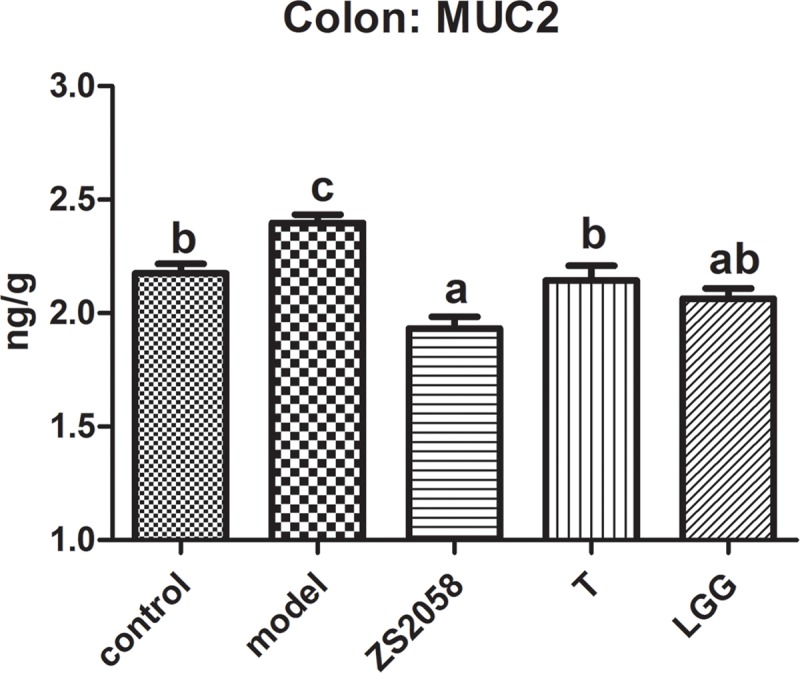
Effects of ZS2058 and LGG on MUC2 levels in the mouse colon. Colons from treated mice were collected and homogenized in cold PBS, and Mucin 2 (MUC2) levels in the homogenates were measured. Data of different groups were analyzed by one-way ANOVA. Data from different groups were considered statistically different at a *p* < 0.05, as indicated by different superscript letters.

A mucus layer covers the intestinal surfaces to form a physiological barrier that excludes luminal bacteria. Several mucins form the gel-forming glycoprotein component of this barrier, of which MUC2 has been identified as the major contributor to the colonic mucus layer (Kumar et al., 2017). As shown in Figure 2, infection with *S.* Typhimurium SL1344 significantly increased the colonic MUC2 levels. However, pretreatment with LGG reduced the production of MUC2 (2.06 ng/g) to a level comparable with that in the uninfected group (2.06 ng/g). However, ZS2058 pretreatment significantly reduced the MUC2 level relative to the uninfected and model groups (1.93 ng/g) (Figure 2). In summary, ZS2058 more effectively reduced MUC2 production in *Salmonella*-infected mice.

#### ZS2058 More Strongly Promoted Activity Through the IL-23/IL-22 and IL-23/IL-17 Axes

**FIGURE 3:**
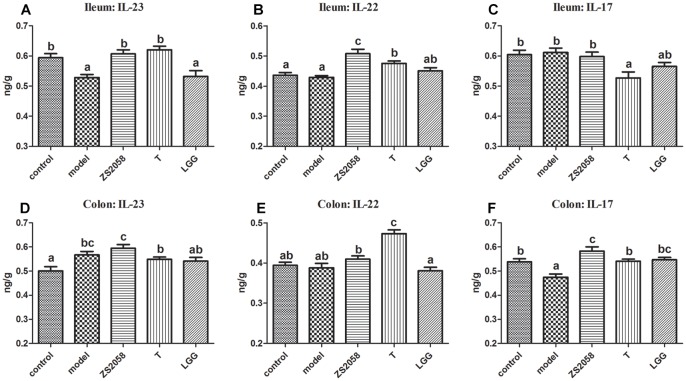
Pretreatment with *L. plantarum* ZS2058 promoted IL-23/IL-22 and IL-23/IL-17 axes. Ilea and colons were homogenized in cold 0.01 M PBS. Subsequently, interleukin (IL)-23 **(A,D)**, IL-22 **(B,E),** and IL-17 levels **(C,F)** in the homogenates were measured. Data of different groups were analyzed by one-way ANOVA. Data from different groups were considered statistically different at a *p* < 0.05, as indicated by different superscript letters.

IL-23 is a critical cytokine associated with host innate immune responses against *Salmonella*. This cytokine can induce IL-17 and IL-22, which are involved in the rapid response to infectious agents (Valeri and Raffatellu, 2016). In the ileum, IL-23 levels decreased significantly in response to infection (0.528 vs. 0.594 ng/g in the uninfected group; Figure 3A), whereas ZS2058 pretreatment restored IL-23 levels in this organ (0.607 ng/g, Figure 3A). By contrast, SL1344 infection had no significant effects on the levels of IL-22 (Figure 3B) and IL-17 (Figure 3C). Both ZS2058 and T (Figure 3B) significantly promoted IL-22 production, whereas IL-17 expression was significantly decreased by T (Figure 3C) and slightly decreased by LGG (Figure 3C). These results demonstrate the ability of ZS2058 to promote the IL23/IL-22 axis in the mouse ileum.

In the colon, both *Salmonella* infection and ZS2058 pretreatment significantly increased the levels of IL-23, whereas LGG had no significant effect (Figure 3D). Regarding IL-22, the ZS2058- and LGG-treated mice, respectively, exhibited a slight increase and slight decrease (non-significant) in the levels of this cytokine (Figure 3E) when compared with PBS-treated and infected mice. Regarding IL-17, *S.* Typhimurium SL1344 infection significantly reduced the expression of this cytokine in the colon (Figure 3F). However, ZS2058 significantly increased the colonic level of IL-17 relative to the control group, whereas LGG only restored this cytokine to a comparable level with the control (Figure 3F). Our results demonstrate that ZS2058 more strongly promotes the IL-23/IL-17 axis in the mouse colon.

### LGG More Effectively Alleviated Gut Inflammation

**FIGURE 4:**
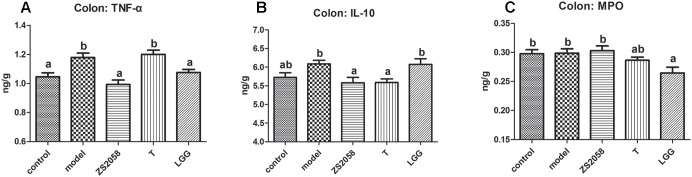
Effects of *L. plantarum* ZS2058 and *L. rhamnosus* GG (LGG) on gut inflammation. Colons were collected from treated mice and homogenized with cold PBS. Tumor necrosis factor (TNF)-α **(A)**, interleukin (IL)-10 **(B),** and myeloperoxidase (MPO) **(C)** levels were measured in the homogenates. Means of different groups were considered statistically different at a *p* < 0.05 by analysis of one-way ANOVA, and indicated by different superscript letters.

Pathogenic infections usually cause gut inflammation, which can be detected by changes in the cytokine profile. As shown in Figure 4A, the levels of the pro-inflammatory cytokine TNF-α in the mice colon increased significantly in response to infection, but were significantly reduced by pretreatment with both ZS2058 and LGG. The levels of IL-10, an important anti-inflammatory cytokine, were slightly increased by infection (Figure 4B) and significantly reduced by ZS2058, but maintained at a comparable level with that in infected mice by LGG (Figure 4B). The level of MPO, a marker of neutrophil infiltration during inflammation, was not significantly affected by infection or pretreatment with ZS2058 or T, but was significantly reduced by LGG (Figure 4C). In summary, only LGG reduced the levels of TNF-α in the colon while maintaining IL-10 and significantly reducing MPO (Figure 4C). These results suggest that LGG more effectively alleviates gut inflammation.

### ZS2058 and LGG Restored IFN-γ Through Different Pathways

**FIGURE 5:**
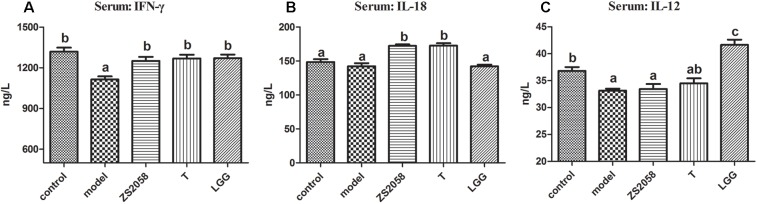
*L. plantarum* ZS2058 and *L. rhamnosus* GG (LGG) restored interferon (IFN)-γ production via different pathways. Interferon (IFN)-γ **(A)**, interleukin (IL)-18 **(B)**, and IL-12 **(C)** levels were measured in sera collected from mice at the time of sacrifice. Means of different groups were considered statistically different at a *p* < 0.05 by analysis of one-way ANOVA, and indicated by different superscript letters.

IFN-γ, a characteristic cytokine in a Th1-type response, may play a major role in enhancing the anti-bacterial activities of macrophages. An *in vivo* study found that IFN-γ deficiency led to an increased splenic *Salmonella* load and decreased survival rate in mice, while treatment with IFN**-γ** prevented the deterioration associated with infection (Eckmann and Kagnoff, 2001). As shown in Figure 5A, the uninfected mice in the control group had a serum IFN-γ level of 1319.4 ng/L. This level decreased significantly to 1114.9 ng/L in response to *S.* Typhimurium SL1344 infection. However, pretreatment with ZS2058 (1250.4 ng/L) or LGG (1271.0 ng/L) restored the IFN-γ levels to comparable with those in the control group (Figure 5A). We subsequently evaluated the levels of IL-12 and IL-18, which induce the production of IFN-γ. Notably, ZS2058 significantly increased the production of IL-18 (Figure 5B) while LGG significantly increased the production of IL-12 (Figure 5C), indicating that these probiotic strains restored IFN-γ levels through distinct pathways.

## Discussion

**FIGURE 6:**
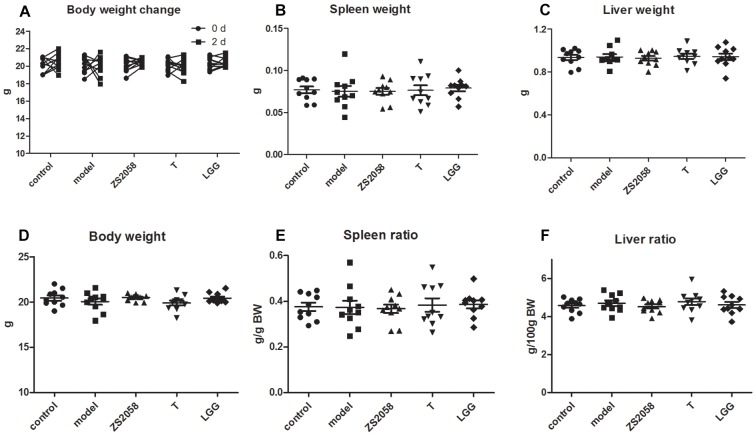
Mice did not exhibit typical symptoms of salmonellosis during the early stage of infection. Mice body weight **(A–D)** were measured immediately after infection and at 2 days post-infection. Spleen **(B)**, and liver **(C)** weight were measured at sacrifice; spleen ratio **(E)**, and liver ratio **(F)** were calculated.

Globally, salmonellosis is associated with high rates of morbidity, hospitalization, and mortality. A loss of body weight, splenomegaly and hepatomegaly are all typical symptoms of salmonellosis. In this study, we found that infected mice did not exhibit any of these symptoms (Figure 6) during the early stage of infection (2 days post-infection), indicating that *S.* Typhimurium SL1344 did not cause severe systemic infection at this time point.

As noted above, CRP is a useful diagnostic marker of infection. In effect, CRP can be produced in many types of inflammation (Ridker, 2003). We observed that the serum CRP level increased significantly in response to *S.* Typhimurium SL1344 infection but decreased in mice that were pretreated with ZS2058 or LGG. We previously reported that *L. plantarum* ZS2058 and *L. rhamnosus* GG, but not *L. plantarum* T, could significantly reduce *Salmonella*-related deaths in a murine model. Our results provide further evidence of the preventive effects of ZS2058 and LGG against *Salmonella* infection. The reduced levels of CRP in infected mice treated with both probiotic strain suggests that the inflammatory responses are alleviated systemically. Although LGG appeared to more strongly alleviate gut inflammation, ZS2058 appeared to be a more effective regulator of systemic inflammation resulting from *Salmonella*.

Following oral infection, pathogens pass through the gastrointestinal tract, where they colonize, survive, replicate and initiate the invasion process. In the gut lumen, the host microbiota plays a crucial role in the inhibition of invasive pathogens through processes including the fermentation of certain carbohydrates to produce SCFAs. In an analysis of the fecal SCFA contents, we found that after a 10-d treatment, only ZS2058 led to a significant increase in the PA level (Table 1). However, ZS2058, T and LGG all tended to increase the levels of acetic acid, PA and butyric acid (Table 1). PA is reported to alter the expression of *S.* Typhimurium genes associated with invasiveness (Lawhon et al., 2002), and to reduce *Salmonella* colonization in the gastrointestinal tract (Levison, 1973). *In vivo* studies have shown that SCFAs promote host defense against *Salmonella* and reduce pathogen loads in the intestinal contents (Sunkara et al., 2011). Accordingly, the ability of ZS2058 to promote the production of PA might contribute to the preventive effects of this probiotic *in vivo*. Increased levels of SCFAs, such as PA, might comprise an important mechanism by which probiotics mediate anti-*Salmonella* functions. Although treatment with lactobacilli might alter the profile or increase the abundance of certain species of host microbiota, a limited substrate might not allow excessive changes in SCFAs. Additional supplementation with substrates, especially prebiotics which are mostly fermented in the colon, might be an effective way to promote the production of SCFAs.

Besides the above-listed effects, SCFAs also contribute to gut immune homeostasis. For example, butyrate can modify the production of II-12 and IL-23 (Berndt et al., 2012). IL-23 induces the production of IL-22 and IL-17 in several cell types, including Th17 cells and NKT cells. In turn, IL-22 can induce the expression of iNOS, the siderophore lipocalin-2 and MUC4 (Raffatellu et al., 2009), while IL-17 recruits neutrophils that play a crucial role in host defense against extracellular bacteria (Santos et al., 2009). We found that in the ileum, LGG treatment did not significantly affect the IL-23/IL-22 (Figure 3A,B) and IL-23/IL-17 (Figure 3A,C) axes when compared with the *Salmonella*-infected group, whereas ZS2058 had a stronger positive effect on the IL-23/IL-22 (Figure 3A,B) axis when compared with T. LGG-treated mice exhibited slight, non-significant changes in the colon IL-23/IL-22 (Figure 3D,E) axis, while ZS2058 promoted the IL-23/IL-22 (Figure 3D,E) and IL-23/IL-17 (Figure 3D,F) axes. In summary, when compared with LGG, ZS2058 more strongly promoted the IL-23/IL-22 and IL-23/IL-17 axes in the host intestines. These axes might act synergistically to enhance host defense and promote pathogen clearance.

Although the host implements various anti-infection measures, *Salmonella* develops survival strategies such as unique respiration (Winter et al., 2010) and self-destructive cooperation (Ackermann et al., 2008). Consequently, surviving pathogens in the gut lumen adhere to the mucosal surface and initiate invasion. *Salmonella* attaches to the mucosal surface by binding to the mannose residues of glycoproteins with assistance from type 1 fimbriae in a process required for colonization of the host intestines (Erbslöh, 2013). As shown in Figure 2, the significant increase in MUC2 in response to *Salmonella* might contribute to this adhesion process. Compared with LGG, ZS2058 more efficiently reduced MUC2 production (Figure 2) in the colon, which might reduce the number of binding sites for *Salmonella*, thus discouraging adhesion and colonization and reducing the risks of invasion.

Taken together, the results listed above suggest that compared to LGG, ZS2058 more effectively reduces the pathogenicity of *Salmonella* in the intestinal phase. Therefore, pathogen control in the host gastrointestinal tract is required to prevent the further development of systemic infection and sepsis. Following intestinal infection, exogenous pathogens trigger a series of local inflammatory responses. Inflammation is a double-edged sword: an appropriate reaction favors the host’s defense against infection, whereas an excessive response may cause unnecessary tissue damage. In the gut, inflammation was reported to provide a respiratory electron acceptor for *Salmonella* that would give it an advantage over the host microbiota (Winter et al., 2010). As shown in Figure 4A, *Salmonella* infection significantly increased the production of TNF-α in the mouse colon. This strong promoter of inflammatory responses suggests a tendency of *Salmonella* to provoke inflammation. LGG was previously reported to down-regulate TNF-α production *in vivo* (Mirpuri et al., 2012), and our observations with LGG and ZS2058 in the present study were consistent with those earlier findings (Figure 4A). Notably, Tedelind et al. (2007) reported that PA could inhibit LPS-induced TNF-α production. Therefore, the increased PA (Table 1) levels in the colon might enhance the ability of ZS2058 to suppress TNF-α production (Figure 4A).

IL-10 is an anti-inflammatory Th2 cytokine that primarily inhibits the production of inflammatory cytokines by innate cells. During infection, IL-10 is required to avoid excessive immune response and prevent development of colitis (Howes et al., 2014). The slight increase in IL-10 (Figure 4B) production in the colons of infected mice might indicate a strategy by which the host controls local inflammatory responses. Notably, IL-10 production in the colons of infected mice was suppressed by ZS2058 and maintained by LGG (Figure 4B), suggesting that the latter probiotic might better control inflammation in the infected gut. We also evaluated the production of MPO, an indicator of neutrophil filtration and colitis severity, in the colons of infected mice. As shown in Figure 4C, *Salmonella* infection and pretreatment with ZS2058 or T had no significant effect on MPO production in the colon. Consistent with a previous report in which LGG reduced MPO production in the lungs (Li et al., 2009), this study revealed a reduction in colonic MPO in mice treated with LGG (Figure 4C). In a murine model, LGG exacerbated the development of DSS-induced colitis and caused animal death (Mileti et al., 2009). However, a clinical study of patients with ulcerative colitis suggests that LGG can effectively and safely maintain remission (Zocco et al., 2006). Based on this study, we suggest that LGG is better able to alleviate gut inflammation during *Salmonella* infection. On one hand, these effects reduced the growth and competitive advantages of *Salmonella* vs. the gut microbiota in an inflammatory environment. On the other hand, excessive tissue damage was averted by the restriction of inflammatory responses.

After crossing the intestinal barrier, salmonellae are phagocytized by host immune cells such as macrophages. The bacteria replicate within these cells and are transported to other internal organs and into the blood (Broz et al., 2012), leading some invasive bacteria to shift from the intestinal phase to a systemic phase of infection. At this stage of infection, the *in vivo* response involves Th1-inducing cytokines that protect against *Salmonella* infection (Mizuno et al., 2003). Of these cytokines, IFN-γ is among the most powerful first-line defense agents against *Salmonella*. IFN-γ limits the availability of iron, withdraws iron from intracellular *Salmonella* (Nairz et al., 2008) and is reported to promote the intracellular killing of bacteria by activating neutrophils and macrophages (Van De Veerdonk et al., 2011). A reduced level of IFN-γ (Figure 5A) in response to infection might enhance the intracellular survival of *Salmonella* and contribute to the development of systemic infection. Pretreatment with ZS2058, T or LGG restored the production of IFN-γ (Figure 5A), which suggests that these lactobacillus strains help to promote the clearance of intracellular bacteria. As mentioned, IL-18 and IL-12 both induce IFN-γ production. Our analyses found that both ZS2058 and T restored IFN-γ production through an IL-18-dependent pathway (Figure 5B), whereas LGG induced the same effect through an IL-12- dependent pathway (Figure 5C). These results demonstrate that the similar effects of probiotics against pathogenic invasion can be mediated by different mechanisms.

**FIGURE 7:**
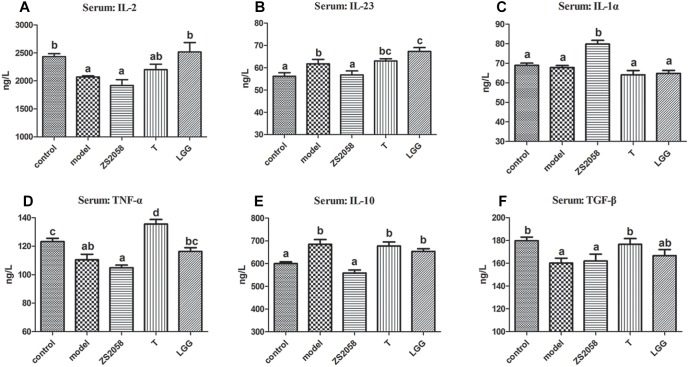
Different effects of *L. plantarum* ZS2058 and *L. rhamnosus* GG (LGG) on the cytokine profiles in sera from infected mice. Sera were collected from mice sacrificed at 2 days post-infection and subjected to ELISAs to detect IL-2 **(A)**, IL-23 **(B)**, IL-1α **(C)**, TNF-α **(D)**, IL-10 **(E)**, and TGF-β **(F)**. Means of different groups were considered statistically different at a *p* < 0.05 by analysis of one-way ANOVA, and indicated by different superscript letters.

Based on the different effects of ZS2058 and LGG on IL-18 and IL-12 production, we hypothesized that these probiotics might modify host immune responses toward pathogens via distinct mechanisms. Our analysis of several cytokines involved in host defense against infection revealed distinct cytokine profiles (Figure 7) in mice pretreated with ZS2058, compared to those pretreated with LGG. Although we did not definitively determine the functions of these modifications in the development and prevention of salmonellosis, we were able to demonstrate the distinct immunoregulatory effects of ZS2058 and LGG to mediate their similar abilities to prevent *Salmonella* infection *in vivo*.

In this study, the previously reported preventive effects of ZS2058 and LGG against *Salmonella* were further verified by the observation of reduced CRP levels in infected mice. We additionally investigated and compared the mechanisms underlying the effects of these two probiotic strains. We found that ZS2058 significantly increased the fecal PA level, increased the colonic MUC2 level and promoted the IL-23/IL-22 and IL-23/IL-17 axes. Our results suggest that compared to LGG, ZS2058 more efficiently reduces the pathogenicity of *Salmonella*. An analysis of the cytokines in the infected mouse colon revealed that LGG more effectively alleviated gut inflammation. Both probiotics restored the production of IFN-γ, a strong promoter of intracellular bacteria clearance, albeit by distinct pathways. Our results therefore demonstrated that ZS2058 and LGG use different mechanisms to exhibit similar anti-*Salmonella* effects in a murine model.

## Author Contributions

JL designed the study, performed research, analyzed data, and prepared the manuscript. ZG supervised the study and revised the manuscript. FS performed research and interpreted results. HZ, JZ, and WC contributed to scientific idea and procedure designing of the study.

## Conflict of Interest Statement

The authors declare that the research was conducted in the absence of any commercial or financial relationships that could be construed as a potential conflict of interest.

## References

[B1] Ackermann M. Stecher B. Freed N. E. Songhet P. Hardt W. D. Doebeli M. (2008). Self-destructive cooperation mediated by phenotypic noise. *Nature* 454 987–990. 10.1038/nature07067 18719588

[B2] Alshawabkeh K. Tabbaa M. J. (2002). Using dietary propionic acid to limit *Salmonella* gallinarum colonization in broiler chicks. *Asian-Australas. J. Anim. Sci.* 15 243–246. 10.5713/ajas.2002.243

[B3] Berndt B. E. Zhang M. Owyang S. Y. Cole T. S. Wang T. W. Luther J. (2012). Butyrate increases IL-23 production by stimulated dendritic cells. *Am. J. Physiol. Gastrointest. Liver Physiol.* 303 G1384-G1392. 10.1152/ajpgi.00540.2011 23086919 PMC3532546

[B4] Broz P. Ohlson M. B. Monack D. M. (2012). Innate immune response to *Salmonella* typhimurium, a model enteric pathogen. *Gut Microbes* 3 62–70. 10.4161/gmic.19141 22198618 PMC3370950

[B5] Castillo N.A. De Leblanc A.D. Galdeano C.M. Perdigon G. (2012). Probiotics: an alternative strategy for combating salmonellosis immune mechanisms involved. *Food Res. Int.* 45 831–841. 10.1016/j.foodres.2011.04.031

[B6] De Keersmaecker S. C. J. Verhoeven T. L. A. Desair J. Marchal K. Vanderleyden J. Nagy I. (2006). Strong antimicrobial activity of *Lactobacillus rhamnosus* GG against *Salmonella* typhimurium is due to accumulation of lactic acid. *Fems Microbiol. Lett.* 259 89–96. 10.1111/j.1574-6968.2006.00250.x 16684107

[B7] Eckmann L. Kagnoff M. F. (2001). Cytokines in host defense against *Salmonella*. *Microbes Infect* 3 1191–1200. 10.1016/S1286-4579(01)01479-4 11755407

[B8] Erbslöh V. (2013). *Methods and Technologies to Reduce or Prevent Salmonella Infection Using Feed Additives in Poultry and Swine Production.* Gödöllõ: Szent István University.

[B9] Howes A. Stimpson P. Redford P. Gabrysova L. O’garra A. (2014). *Interleukin-10: Cytokines in Anti-Inflammation and Tolerance.* Springer, Berlin 10.1007/978-4-431-54442-5_13

[B10] Hudault S. Lievin V. Bernetcamard M. F. Servin A. L. (1997). Antagonistic activity exerted in vitro and in vivo by *Lactobacillus casei* (strain GG) against *Salmonella* typhimurium C5 infection. *Appl. Environ. Microbiol.* 63 513–518. 9023930 10.1128/aem.63.2.513-518.1997 PMC168342

[B11] Kemgang T. S. Kapila S. Shanmugam V. P. Reddi S. Kapila R. (2016). Fermented milk with probiotic Lactobacillus rhamnosus S1K3 (MTCC5957) protects mice from *salmonella* by enhancing immune and nonimmune protection mechanisms at intestinal mucosal level. *J. Nutri. Biochem.* 30 62–73. 10.1016/j.jnutbio.2015.11.018 27012622

[B12] Kumar M. Kissoon-Singh V. Coria A. L. Moreau F. Chadee K. (2017). Probiotic mixture VSL#3 reduces colonic inflammation and improves intestinal barrier function in Muc2 mucin-deficient mice. *Am. J. Physiol. Gastrointest. Liver Physiol.* 312 G34-G45. 10.1152/ajpgi.00298.2016 27856417

[B13] Lawhon S. D. Maurer R. Suyemoto M. Altier C. (2002). Intestinal short-chain fatty acids alter *Salmonella* typhimurium invasion gene expression and virulence through BarA/SirA. *Mol. Microbiol.* 46 1451–1464. 10.1046/j.1365-2958.2002.03268.x 12453229

[B14] Levison M. E. (1973). Effect of colon flora and short-chain fatty acids on growth in vitro of *Pseudomonas* aeruginsoa and *Enterobacteriaceae*. *Infect. Immun.* 8 30–35. 4198102 10.1128/iai.8.1.30-35.1973 PMC422805

[B15] Li N. Russell W. M. Douglas-Escobar M. Hauser N. Lopez M. Neu J. (2009). Live and heat-killed Lactobacillus rhamnosus GG: effects on proinflammatory and anti-inflammatory cytokines/chemokines in gastrostomy-fed infant rats. *Pediatr. Res.* 66 203–207. 10.1203/PDR.0b013e3181aabd4f 19390478

[B16] Li X. Xu Q. Jiang T. Fang S. Wang G. Zhao J. (2016). A comparative study of the antidiabetic effects exerted by live and dead multi-strain probiotics in the type 2 diabetes model of mice. *Food Funct.* 7 4851–4860. 10.1039/c6fo01147k 27812581

[B17] Liu J. Hu D. Chen Y. Huang H. Zhang H. Zhao J. (2018). Strain-specific properties of Lactobacillus plantarum for prevention of *Salmonella* infection. *Food Funct.* 9 3673–3682. 10.1039/c8fo00365c 29956713

[B18] McDade T. W. Rutherford J. N. Adair L. Kuzawa C. (2008). Adiposity and pathogen exposure predict C-reactive protein in Filipino women. *J. Nutr.* 138 2442–2447. 10.3945/jn.108.092700 19022970 PMC2801568

[B19] Mileti E. Matteoli G. Iliev I. D. Rescigno M. (2009). Comparison of the immunomodulatory properties of three probiotic strains of Lactobacilli using complex culture systems: prediction for in vivo efficacy. *PLoS One* 4:e7056. 10.1371/journal.pone.0007056 19756155 PMC2738944

[B20] Mirpuri J. Sotnikov I. Myers L. Denning T. L. Yarovinsky F. Parkos C. A. (2012). Lactobacillus rhamnosus (LGG) regulates IL-10 signaling in the developing murine colon through upregulation of the IL-10R2 receptor subunit. *PLoS One* 7:e51955. 10.1371/journal.pone.0051955 23272193 PMC3525658

[B21] Mizuno Y. Takada H. Nomura A. Jin C. H. Hattori H. Ihara K. (2003). Th1 and Th1-inducing cytokines in *Salmonella* infection. *Clin. Exp. Immunol.* 131 111–117. 10.1046/j.1365-2249.2003.02060.x 12519393 PMC1808588

[B22] Nairz M. Fritsche G. Brunner P. Talasz H. Hantke K. Weiss G. (2008). Interferon-gamma limits the availability of iron for intramacrophage *Salmonella* typhimurium. *Eur. J. Immunol.* 38 1923–1936. 10.1002/eji.200738056 18581323

[B23] Neill M. A. Opal S. M. Heelan J. Giusti R. Cassidy J. E. White R. (1991). Failure of ciprofloxacin to eradicate convalescent fecal excretion after acute salmonellosis: experience during an outbreak in health care workers. *Ann. Intern. Med.* 114 195–199. 10.7326/0003-4819-114-3-195 1898630

[B24] Pascual M. Hugas M. Badiola J. I. Monfort J. M. Garriga M. (1999). *Lactobacillus salivarius* CTC2197 prevents *Salmonella* enteritidis colonization in chickens. *Appl. Environ. Microbiol.* 65 4981–4986. 10543812 10.1128/aem.65.11.4981-4986.1999 PMC91670

[B25] Raffatellu M. George M. D. Akiyama Y. Hornsby M. J. Nuccio S. P. Paixao T. A. (2009). Lipocalin-2 resistance confers an advantage to *Salmonella enterica* serotype Typhimurium for growth and survival in the inflamed intestine. *Cell Host. Microbe* 5 476–486. 10.1016/j.chom.2009.03.011 19454351 PMC2768556

[B26] Rajashekara G. Haverly E. Halvorson D. A. Ferris K. E. Lauer D. C. Nagaraja K. V. (2000). Multidrug-resistant *Salmonella* Typhimurium DT104 in poultry. *J. Food Protect.* 63 155–161. 10.4315/0362-028x-63.2.155 10678417

[B27] Ridker P. M. (2003). Clinical application of C-reactive protein for cardiovascular disease detection and prevention. *Circulation* 107 363–369. 10.1161/01.CIR.0000053730.47739.3C 12551853

[B28] Santos R. L. Raffatellu M. Bevins C. L. Adams L. G. Tukel C. Tsolis R. M. (2009). Life in the inflamed intestine, *Salmonella* style. *Trends Microbiol.* 17 498–506. 10.1016/j.tim.2009.08.008 19819699 PMC3235402

[B29] Sunkara L. T. Achanta M. Schreiber N. B. Bommineni Y. R. Dai G. Jiang W. (2011). Butyrate enhances disease resistance of chickens by inducing antimicrobial host defense peptide gene expression. *PLoS One* 6:e27225. 10.1371/journal.pone.0027225 22073293 PMC3208584

[B30] Tanner S. A. Chassard C. Rigozzi E. Lacroix C. Stevens M. J. A. (2016). Bifidobacterium thermophilum RBL67 impacts on growth and virulence gene expression of *Salmonella enterica* subsp enterica serovar Typhimurium. *BMC Microbiology* 16.46 10.1186/s12866-016-0659-x 26988691 PMC4797131

[B31] Tedelind S. Westberg F. Kjerrulf M. Vidal A. (2007). Anti-inflammatory properties of the short-chain fatty acids acetate and propionate: a study with relevance to inflammatory bowel disease. *World J. Gastroenterol.* 13 2826–2832. 10.3748/wjg.v13.i20.2826 17569118 PMC4395634

[B32] Valeri M. Raffatellu M. (2016). Cytokines IL-17 and IL-22 in the host response to infection. *Pathog. Dis.* 74:ftw111. 10.1093/femspd/ftw111 27915228 PMC5975231

[B33] Van De Veerdonk F. L. Netea M. G. Dinarello C. A. Joosten L. A. (2011). Inflammasome activation and IL-1beta and IL-18 processing during infection. *Trends Immunol.* 32 110–116. 10.1016/j.it.2011.01.003 21333600

[B34] Winter S. E. Thiennimitr P. Winter M. G. Butler B. P. Huseby D. L. Crawford R. W. (2010). Gut inflammation provides a respiratory electron acceptor for *Salmonella*. *Nature* 467 426–429. 10.1038/nature09415 20864996 PMC2946174

[B35] Yeung C.Y. Chiau J.S.C. Chan W.T. Jiang C.B. Cheng M.L. Liu H.L. (2013). In vitro prevention of *Salmonella* lipopolysaccharide-induced damages in epithelial barrier function by various lactobacillus strains. *Gastroenterol. Res. Pract.* 3:973209. 10.1155/2013/973209 23840201 PMC3690232

[B36] Yoshimi N. Matsunaga K. Katayama M. Yamada Y. Kuno T. Qiao Z. (2001). The inhibitory effects of mangiferin, a naturally occurring glucosylxanthone, in bowel carcinogenesis of male F344 rats. *Cancer Lett.* 163 163–170. 10.1016/S0304-3835(00)00678-9 11165750

[B37] Zocco M. A. Dal Verme L. Z. Cremonini F. Piscaglia A. C. Nista E. C. Candelli M. (2006). Efficacy of lactobacillus GG in maintaining remission of ulcerative colitis. *Aliment Pharmacol. Ther.* 23 1567–1574. 10.1111/j.1365-2036.2006.02927.x. 16696804

